# Investigation of Choroidal Circulation Hemodynamics Using Laser Speckle Flowgraphy After Periocular Skin Warming

**DOI:** 10.7759/cureus.75118

**Published:** 2024-12-04

**Authors:** Chihiro Ujita, Yuki Hashimoto, Kota Noguchi, Nao Nakamura, Miki Yoshimura, Sarari Ichiki, Moka Uehara, Aimi Nakazaki, Takanori Taniguchi, Takeshi Yoshitomi

**Affiliations:** 1 Orthoptics, Fukuoka International University of Health and Welfare, Fukuoka, JPN; 2 Physical Therapy, Fukuoka International University of Health and Welfare, Fukuoka, JPN

**Keywords:** choroidal circulation hemodynamics, laser speckle flowgraphy, mean blur rate, parasympathetic activity, systemic circulation

## Abstract

Purpose: The aim of the current study was to evaluate changes in choroidal circulation hemodynamics after periocular skin warming at 40°C using laser speckle flowgraphy (LSFG).

Methods: Twenty-four right eyes of 24 healthy participants were included. Changes in choroidal circulation hemodynamics were determined using LSFG to evaluate the mean blur rate (MBR) of the macula, which represents choroidal blood flow velocity. Systolic blood pressure (SBP), diastolic blood pressure (DBP), mean blood pressure (MBP), heart rate (HR), ocular perfusion pressure (OPP), and subfoveal choroidal thickness (SCT) were also measured, and sympathetic nerve status was assessed via salivary α-amylase (sAA) activity at baseline and after periocular skin warming.

Results: Immediately after warming, SBP, DBP, MBP, HR, OPP, sAA activity, and MBR were significantly lower than baseline. SCT was significantly increased.

Conclusion: In the normal eye, the decrease in sympathetic nerve activity induced by the relaxing effects of periocular skin warming reduces systemic circulatory activity and choroidal circulatory dynamics.

## Introduction

Thermotherapy can reduce pain and anxiety via physical effects such as increased skin temperature and blood flow, and psychological effects such as pain relief and comfort [1,2]. Sawada et al. [2] reported that 42°C thermal stimulation improved subjective symptoms such as muscle stiffness and fatigue compared to no stimulation, and that recipients of the thermal stimulation felt refreshed. An increase in nasal mucosal temperature after foot warming has been reported to be mediated by a neural reflex due to the loss of sympathetic activation in the nasal vascular system, and long-acting parasympathetic mediators may also be contributary [3].

Several studies investigating periocular skin warming have shown that it reduces sympathetic activity and increases parasympathetic activity [4-9], promotes sleep onset in normal subjects and in patients with sleep difficulties or insomnia [5-7], and has a positive effect on subjective wellbeing upon awakening [8]. It has also been associated with increases in subjective and objective regulation, and a concomitant improvement in near visual acuity [9].

There are changes in choroidal circulation hemodynamics and choroidal morphology after immersion of the foot in warm water. Parasympathetic nerve activity induced by warming stimuli decreases choroidal blood flow velocity and increases choroidal thickness in response to reduced systemic circulatory activity, including systolic blood pressure (SBP), diastolic blood pressure (DBP), and mean blood pressure (MBP) [10,11]. However, to date, no studies have evaluated choroidal circulation hemodynamics after periocular skin warming.

Therefore, we aimed to clarify the effects of periocular thermotherapy on the autonomic nervous system and changes in systemic and choroidal circulating hemodynamics and morphology by the application of 40°C warming to the periocular skin. To the best of our knowledge, this is the first study to report changes in mean blur rate (MBR) after periocular skin warming.

## Materials and methods

Participants

This study included 24 right eyes of 24 healthy young Japanese adults recruited via non-probability sampling with voluntary responses. All participants had best corrected visual acuity (BCVA) of 20/20 or better, no abnormalities in fundus findings, no cardiovascular or ophthalmologic diseases, and were not using ophthalmic or systemic medications. All participants underwent color fundus photography (TRC-NW 200; TOPCON, Co., Ltd., Japan). Their refractive error (ARK-1a; NIDEK, Co., Ltd., Kyoto, Japan), axial length (AL-Scan; NIDEK Co., Ltd., Kyoto, Japan), BCVA, intraocular pressure (IOP) (NT-530; NIDEK Co., Ltd., Kyoto, Japan), blood pressure (BP) and heart rate (HR) (HEM-1021; OMRON Healthcare Co., Ltd., Muko, Japan), and salivary α-amylase (sAA) activity (Saliva Amylase Monitor; NIPRO Co., Ltd., Osaka, Japan) were measured. They also underwent enhanced depth imaging optical coherence tomography (EDI-OCT) (RS-3000 Advance 2; NIDEK Co., Ltd., Kyoto, Japan) and laser speckle flowgraphy (LSFG) (Softcare Ltd., Fukuoka, Japan).

Study design

This prospective study adhered to the tenets of the Declaration of Helsinki, and all participants provided written informed consent. The study protocol was approved by the Ethics Committee of Fukuoka International University of Health and Welfare (approval ID: 21-fiuhw-003). Participants were recruited from June 1, 2024, to August 1, 2024. The examinations were conducted in the following order: LSFG, BP and HR measurement, EDI-OCT, IOP measurement, and sAA activity measurement. These had a duration of 90 s, 60 s, 15 s, and 30 s, respectively. They were all performed between 11:00 and 16:00 and completed within 5 min.

Periocular skin warming

All participants were tested in a uniform and comfortable environment, with the room temperature set at 24°C ± 1°C and humidity at 47% ± 3% [10-14]. The temperature and humidity were measured near the participants. The illumination in the room during measurement was set to 1 lx. Thermal sheets (MegRhythm^Ⓡ^; Kao, Japan) emitting moist heat of approximately 40°C were used as eye masks for periocular skin warming. The thermal sheets are disposable and become warm immediately after opening, reaching 40℃ after 1 to 2 min. These conditions were same for all participants. The duration of use was set at 20 min, which is the time required to maintain a comfortable temperature, as specified in the user manual. All examinations were performed at baseline and immediately after warming. Each participant was instructed to avoid smoking, exercising, or caffeine intake for at least 2 h before testing, and to rest for 10 min in the examination room.

IOP, hemodynamics, sAA, and EDI-OCT measurements

IOP, SBP, DBP, sAA activity, subfoveal choroidal thickness (SCT), and MBR were evaluated before and immediately after warming. IOP measurements were performed using a non-contact tonometer. Choroidal thickness was measured manually by taking 120 additional B-scan images with a scan length of 12.0 mm of horizontal cross sections with EDI-OCT and measuring the vertical distance in the subfovea from the lower edge of the retinal pigment epithelium below the boundary between the choroid and the sclera. SCT was considered as the average of the measurements obtained by two examiners (Y.H., K.N.). MBP was calculated from SBP and DBP via the following equation:

 *MBP = DBP + 1/3(SBP - DBP)* (1)

Ocular perfusion pressure (OPP) was calculated from MBP and IOP via the following equation:

*OPP = 2/3MBP - IOP* (2)

LSFG measurements

**Figure 1:**
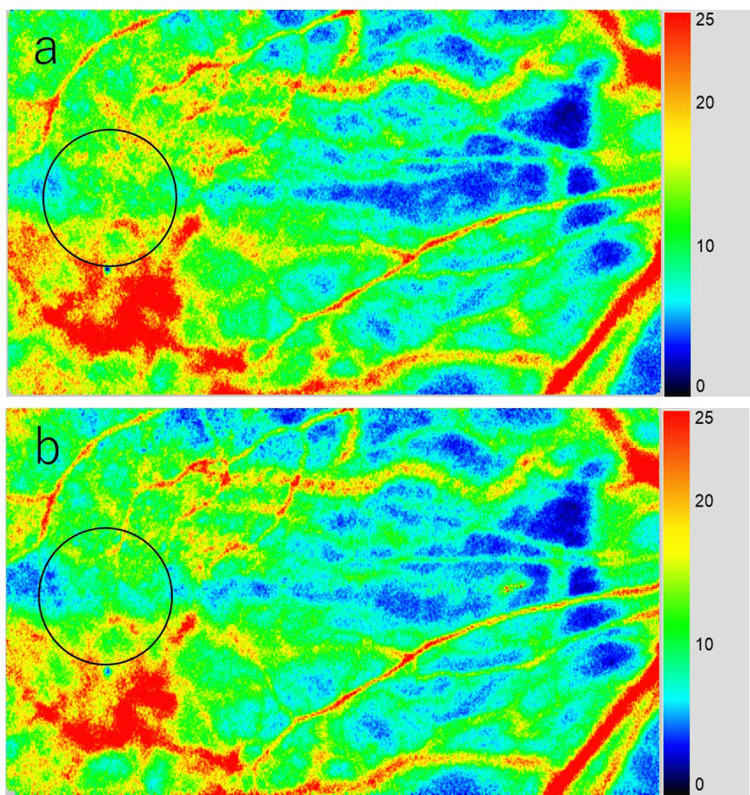
Composite color map of the MBR measured by LSFG. LSFG images at baseline (a) and immediately after periocular skin warming (b) of a participant. After periocular skin warming, MBR was reduced by 18% compared to baseline. MBR, mean blur rate; LSFG, laser speckle flowgraphy.

LSFG was used to measure the hemodynamics of the posterior fundus [14,15]. It uses an 830-nm diode laser to illuminate the fundus and detect moving red blood cells in deeply choroidal vessels [16,17]. Each LSFG measurement was taken three times consecutively at baseline and immediately after periocular skin warming. The laser speckle method has the advantage of allowing for quantitative and repeated examinations; the use of this method to measure ocular blood velocity has been reported to yield reproducible results [15,18]. Changes in choroidal blood flow velocity were assessed by excluding large retinal vessels, so that retinal blood flow velocity was not affected (Figure 1). When healthy individuals were followed up, each circle was automatically set using LSFG Analyzer software (v. 3.0.47; Softcare Ltd., Fukuoka, Japan) at the same site where the circle was set at baseline. To ensure similar conditions for all participants, the circular area for analyzing the macular MBR was set to a size of 250×250 pixels. The position was identified using the fundus photograph. Each MBR was automatically calculated using the software. As MBR is a relative value, the initial baseline value for calculating the change in mean MBR was set at 100% [11,13,19]. The MBR value was the average of three measurements.

Statistical analysis

All results are expressed as means ± the standard deviation. The Wilcoxon signed-rank test was used to assess changes in IOP, SBP, DBP, MBP, OPP, sAA activity, SCT, and MBR. Additionally, multiple regression analysis was conducted. The response variable was the rate of change in MBR, while the explanatory variables included change in IOP, SBP, DBP, MBP, HR, sAA activity, and SCT before and after thermotherapy; and basic information (gender, age, refractive error, and axial length). The Spearman's rank correlation coefficient was used to examine the correlation between the SCT measurements performed by the two operators. For all tests, p <0.05 was considered statistically significant. All statistical analyses were performed using BellCurve for Excel (Social Survey Research Information Co., Ltd., Shinjuku, Japan).

## Results

Twenty-four right eyes of 24 healthy volunteers (18 women and six men) were examined in this study. Their mean age was 21.0 ± 4.1 years (range 18-39 years). The mean refractive error was -2.90 ± 2.50 D (range +1.50 to -7.25 D). The mean axial length was 25.18 ± 1.23 mm (range 22.76 to 27.95 mm).

IOP, hemodynamics, sAA, and SCT

IOP, SBP, DBP, MBP, HR, and sAA activity changes are shown in Table 1. Compared to baseline, immediately after periocular skin warming, there were significant reductions in SBP (p = 0.022), DBP (p < 0.001), MBP (p = 0.001), HR (p < 0.001), and sAA activity (p = 0.009) (Table 1). SCT was significantly increased after periocular warming therapy compared with that of baseline (p < 0.001). IOP did not change significantly (p = 0.166). Additionally, there was a significant positive correlation between the SCT measurements obtained by the two operators before and after thermotherapy (r = 0.971, p < 0.001 and r = 0.963, p < 0.001).

**Table 1:** Changes in ocular biometric parameters and systemic factors at baseline and after the periocular thermotherapy. SD, standard deviation; IOP, intraocular pressure; SBP, systolic blood pressure; DBP, diastolic blood pressure; MBP, mean blood pressure; HR, heart rate; OPP, ocular perfusion pressure; sAA, salivary amylase activity; SCT, subfoveal choroidal thickness; MBR, mean blur rate.

	Baseline (mean ± SD/median)	After the thermotherapy (mean ± SD/median)	p-Value (Wilcoxon signed-rank test)
IOP (mmHg)	13.7 ± 2.4/13.5	13.2 ± 2.2/13.0	0.166
SBP (mmHg)	111.5 ± 11.3/109.8	105.1 ± 11.0/102.8	<0.001
DBP (mmHg)	68.6 ± 9.4/68.8	66.0 ± 9.1/65.5	0.026
MBP (mmHg)	82.9 ± 9.8/82.6	79.1 ± 9.4/78.0	0.001
HR (mmHg)	81.3 ± 10.0/79.3	77.3 ± 9.8/73.8	<0.001
OPP (mmHg)	46.1 ± 6.3/46.3	43.9 ± 6.2/43.0	0.003
sAA (kU/L)	12.9 ± 12.3/9.0	7.1 ± 7.1/4.0	0.009
SCT (µm)	260 ± 79.4/236.3	264 ± 81.0/238.3	<0.001
MBR	13.0 ± 4.6/12.0	10.7 ± 4.0/9.7	<0.001
MBR (%)	100.0 ± 0.0/100	82.7 ± 8.1/81.7	<0.001

LSFG

Changes in MBR are shown in Table 1. The mean macular MBR at baseline was 13.0 ± 4.6, and that immediately after periocular skin warming was 10.7 ± 4.0. Macular MBR significantly decreased by -17.3% ± 8.1% after warming (p < 0.001).

Multiple regression analysis

In the multiple regression analysis, no parameter was a significant explanatory variable (Table 2).

**Table 2:** Results of multiple regression analysis. IOP, intraocular pressure; SBP, systolic blood pressure; DBP, diastolic blood pressure; MBP, mean blood pressure; HR, heart rate; OPP, ocular perfusion pressure; sAA, salivary α-amylase; SCT, subfoveal choroidal thickness; RE, refractive error; AL, axial length.

Variable name	Partial regression coefficient	t-Value	p-Value	Correlation coefficient
IOP	52.682	1.157	0.271	0.228
SBP	-18.639	-0.525	0.609	0.526
DBP	-39.293	-0.552	0.591	0.065
MBP	6.394	-0.081	0.936	0.261
HR	-0.134	-0.356	0.728	-0.282
OPP	77.726	1.144	0.276	0.187
sAA	-0.217	-1.203	0.254	-0.465
SCT	0.152	0.197	0.847	0.091
Sex	0.776	0.133	0.896	-0.078
Age	-0.270	-0.549	0.593	-0.239
RE	-1.667	-1.353	0.203	-0.143
AL	-3.678	-1.274	0.228	-0.120

## Discussion

In this study choroidal blood flow velocity decreased significantly after periocular skin warming at 40°C for 20 min, as did SBP, DBP, MBP, HR, and sAA activity with parasympathetic activity predominant. Contrarily, SCT increased significantly. Decreased sympathetic activity leads to vasodilation and reduced BP [20,21]. The choroid is susceptible to autonomic nervous activity due to its lack of autoregulation. In summer, at night, in the late follicular phase, and a cold pressor test, accompanying a decrease in BP, choroidal blood flow velocity decreases and SCT increases [12,13,19,22-24]. Interestingly, in a previous study [10], SBP, DBP, MBP, OPP, and choroidal blood flow velocity reduced significantly after foot immersion in 40°C water for 60 s, and MBR was significantly positively correlated with SBP, DBP, MBP, and OPP. It has also been reported that choroidal thickness increased significantly in conjunction with significant reductions in SBP, DBP, and MBP after immersion of the foot in 40°C water for 60 s [11]. These changes are in response to parasympathetic nervous activity induced by the comfortable and relaxing heating stimulus of 40°C, which reduces systemic circulatory activity [10,11]. Thus, it is suggested that choroidal blood flow velocity decreases accordingly, increasing choroidal thickness.

Central serous chorioretinopathy (CSC) is associated with increased sympathetic activity, reduced parasympathetic activity, an altered sympathetic-parasympathetic balance, and reduced parasympathetic responsiveness [25]. Autonomic dysfunction in the form of increased sympathetic tone and decreased reactivity is observed in patients with CSC. Inappropriate sympathetic responses during stressor stimulation may be an important factor in the development of CSC [25]. The digestive enzyme, sAA, has been used as an indicator of sympathetic nervous system activity in healthy persons. In a previous report that measured sAA activity in patients with CSC, morning sAA activity was significantly higher in patients with active CSC compared to patients with inactive CSC and compared to healthy control subjects [26]. In previous studies investigating acute CSC, macular MBR increased in the acute phase and decreased in the remission phase as visual function and ocular findings improved, indicating that increased choroidal blood flow velocity associated with sympathetic hyperactivity is a factor in the pathogenesis of acute CSC [27,28]. It is therefore important that future studies directly observe autonomic nervous system activity after periocular skin heating and evaluate whether increasing parasympathetic dominance can prevent the onset of CSC or alleviate ocular symptoms related to sympathetic hyperactivity.

The current study has several limitations. First, the results of this study could be further verified by comparing them with those of a sham group or a cooling stimulus group as controls. Choroidal circulation hemodynamics and morphology were only measured via LSFG and EDI-OCT. To overcome this, OCT angiography to evaluate vascular density and binarization evaluation methods should be used to clarify whether choroidal blood vessels dilate when the parasympathetic nervous system is dominant. Second, the only direct measurement of neural activity in the study was sAA activity. More detailed investigation of relationships between changes in choroidal circulatory dynamics requires the use of electrocardiographic assessment of neural activity and pupillary response assessment. Third, this study included participants with high myopia and a long axial length; however, it is possible that refractive errors and axial length may affect choroidal changes. Fourth, the small sample size in this study is insufficient for interpreting the results of multiple regression analysis. Future studies should investigate choroidal changes in periocular skin warming in more detail using a larger sample size and various refractive errors and axial lengths.

## Conclusions

The study suggests that in healthy individuals, parasympathetic activity induced by periocular skin warming reduces systemic dynamics and choroidal blood flow velocity.
